# Evaluation of Clinical Competence for a Cardiology Residency Program

**DOI:** 10.36660/abc.20190842

**Published:** 2020-01

**Authors:** Sergio Timerman

**Affiliations:** Instituto do Coração (Incor) do Hospital das Clínicas da Universidade de São Paulo - Divisão de Cardiologia Clínica - Centro de Simulação e Time de Resposta Rápida, São Paulo, SP - Brazil

**Keywords:** ST Elevation Myocardial Infarction, Simulation, Internship and Residence, Hospitals, Programs, Medical Competence

In the past decades, medical education, especially in cardiology gratuate programs (CGP), has undergone profound changes, including restrictions on hours of service.^1^ In the reference article “To Err Is Human”,^2^ the Institute of Medicine suggests that nearly 100,000 patients die annually from preventable errors in hospitals, with another one million people with sequelae. This report turned the spotlight on the importance of patient safety with regard to healthcare.^3^ At about the same time, technological progress has outweighed curriculum innovations of EMPGs. As we were trained on the job, the method “see one, do one, and teach one” was common for all services, but as training progresses, procedures become extremely complex, with consequently higher risks. Most cardiology residents remember the first time they performed resuscitation maneuvers, placed transvenous pacemakers, and passed their first Swan-Ganz.^4^ Fortunately, most of these events were completed without complications. However, the level of concern and anxiety experienced regarding patient safety and their competence to perform these tasks is probably as vivid now as the day the procedure was performed. Despite the scarcity of evidence supporting the traditional training learning model,^5,6^ most reviews discussing the potential of simulation-based education (SBE) for healthcare assess evidence that SBE is equivalent to or better than this traditional model.^7-9^

Nowadays, for the recent graduates and candidates for Medical Residency, assessment of clinical skills is an essential step and should be started in their education as a medical student, and should be done by the professor through direct observation of their performance in real situations.^10^ This formative and summative assessment takes different forms, as it assesses students’ clinical competences and quantifies the evolution of their performance based on real-life situations.^11,12^ The study published in this issue, entitled “Clinical Competence in ST-segment Elevation Myocardial Infarction Management by Recently Graduated Physicians Applying for a Medical Residency Program”^13^ aims to analyze the following: skills seen in the interview, physical examination skills, professionalism (ethics), clinical reasoning, orientation skills, efficiency and general clinical competence, pointing out their flaws and successes, making it a good weapon in formative assessment. Simulation training has also been widely adopted in other "high risk" industries. Although comparisons between medicine and aviation are frequent, it is important to recognize that the work performed by doctors differs a lot from that of pilots, so the nature of simulation must also be different. There is considerable focus on medical emergencies and practical procedural skills, but with scope to expand to other areas of care. The contribution of human cognitive performance to patient outcomes is well recognized; possessing the necessary knowledge and technical skills remains essential, but in addition to them, non-technical skills, such as situational awareness and the ability to synthesize information, making decisions and effectively communicating with team members during times of stress and distraction are also essential. And this study was important for this reason.
